# The Effect of Femtosecond Laser Surface Patterns on the Effectiveness of Resin Composite to Zirconia Bonding

**DOI:** 10.3390/jfb14100508

**Published:** 2023-10-11

**Authors:** Majed M. Alsarani, Omar Alsadon, Omar Alageel, Najm Alfrisany, Zeyad Almutairi, Mahmoud A. Al-Gawati, Mayyadah Almozainy

**Affiliations:** 1 Dental Health Department, College of Applied Medical Sciences, King Saud University, Riyadh 12372, Saudi Arabia; oalsadon@ksu.edu.sa (O.A.); oalageel@ksu.edu.sa (O.A.); nalfrisany@ksu.edu.sa (N.A.); 2 Department of Mechanical Engineering, College of Engineering, King Saud University, Riyadh 11421, Saudi Arabia; zaalmutairi@ksu.edu.sa; 3 Department of Physics and Astronomy, College of Science, King Saud University, Riyadh 11451, Saudi Arabia; malgawati@ksu.edu.sa; 4 Department of Restorative Dental Science, College of Dentistry, King Saud University, Riyadh 12371, Saudi Arabia; malmozainy@ksu.edu.sa

**Keywords:** zirconia ceramic, resin composite, enclosed mold shear bond strength (EM-SBS), femtosecond laser, surface treatment

## Abstract

This laboratory study aimed to evaluate the effect of different surface patterns using femtosecond laser treatment on the enclosed mold shear bond strength (EM-SBS) of resin composite to zirconia (ZrO_2_) surfaces and to contrast it with the widely used tribochemical silica coating (TBC) surface conditioning method. A set of fifteen rectangular ZrO_2_ blocks were randomly divided into five groups according to surface pretreatment: Control G_0_—no treatment; G_1_—TBC with silane application; G_2_—femtosecond laser irradiation with horizontal lines 30 µm apart; _G3_—femtosecond laser irradiation with horizontal lines 15 µm apart; and G_4_—femtosecond laser irradiation with cross lines 30 µm apart. The pretreated surfaces were characterized by a surface profilometer, tensiometer and scanning electron microscope. The EM-SBS of resin composite stubs to ZrO_2_ was measured followed by fractographic analysis. The surface roughness and water contact angle were observed to be statistically higher among the femtosecond laser groups compared to the TBC and control groups. The G_4_ group exhibited the highest EM-SBS among all the groups, irrespective of the ageing conditions used. At the end of 5000 thermocycles, G_4_ exhibited EM-SBS of 14.05 ± 4.21 MPa compared to 13.80 ± 3.01 MPa in G_1_ and 5.47 ± 0.97 MPa in G_0_. The two-way ANOVA revealed a significant effect of both study groups and ageing conditions on the EM-SBS (*p* < 0.001). Utilization of femtosecond laser technology holds promise as a potential and alternative mechanical retention approach for enhancing the bonding strength of the resin composite to ZrO_2_.

## 1. Introduction

Contemporary surface treatments utilized for bonding resin composite to zirconia (ZrO_2_) can potentially generate unintended microcracks and surface flaws [1]. ZrO_2_, particularly yttria-stabilized tetragonal ZrO_2_ (Y-TZP), stands out as a ceramic possessing superior mechanical properties compared to other dental ceramics [2]. Moreover, it exhibits commendable chemical and structural stability and offers enhanced esthetics in comparison to metal–ceramic materials [2]. This high-strength ceramic material finds applicability as a substructure for fixed partial dentures supported by either natural teeth or dental implants.

In the challenging oral environment, all dental restorations are subjected to substantial stresses [3]. Achieving a durable bond between materials, such as ZrO_2_ restorations and resin composite cement, presents a significant challenge in prosthetic dentistry [4]. In recent years, extensive research has focused on developing novel preparation techniques aimed at enhancing the bond strength between resin composite and ceramic surfaces. These techniques include airborne-particle abrasion, tribochemical silica coatings (TBC), hydrofluoric acid etching, and neodymium-doped yttrium aluminum garnet (Nd:YAG) laser irradiation [3]. However, to date, TBC is a widely used method for surface pretreatment. 

Several studies investigating the application of laser irradiation for modifying dental ceramics have demonstrated encouraging outcomes [5,6]; Nevertheless, these studies have certain limitations. Yucel et al. [7] and Ersu et al. [8] reported the occurrence of crack formation in ceramics due to thermal damage when utilizing Nd:YAG and CO_2_ lasers, respectively. Additionally, the use of Er:YAG and CO_2_ irradiation on the surface of ZrO_2_ ceramics has been found to result in the formation of microcracks, pits, and melted areas on the ZrO_2_ surface [1]. These observations highlight the potential drawbacks and challenges associated with employing laser irradiation techniques for ceramic modification.

Advancements in laser technologies have led to the introduction of femtosecond lasers capable of producing ultrashort pulses. Within this context, femtosecond laser surface texturing has emerged as an innovative and versatile technology utilizing Nd:YLF for creating surfaces characterized by multimodal roughness and high curvature of textural elements, thereby catering to a wide range of applications [9]. The utilization of ultrashort (femtosecond) laser pulses has proven effective in facilitating precise micromachining of various materials, including ZrO_2_ ceramics while minimizing damage [10]. Femtosecond laser micromachining enables controlled surface roughening of ZrO_2_ with high precision and minimal thermal impact, thereby reducing the presence of residual elements. Furthermore, the resulting surface exhibits enduring characteristics and does not undergo phase transformation. However, the disadvantages associated with this laser system are the cost and processing time [11].

The establishment of a robust and long-lasting bond between resin cement and ZrO_2_ is widely recognized as an essential requirement for clinical success [12]. Consequently, the objectives of this study were: (a) to compare and contrast various surface conditioning methods, in particular TBC and femtosecond laser irradiation on enclosed mold-shear bond strength (EM-SBS) between resin composite and ZrO_2_ substrate, and (b) to analyze the types of failures observed during debonding. The null hypothesis tested posited that the evaluated surface conditioning methods would not exhibit significant differences in terms of the bonding and fractographic analysis of the resin composite to the ZrO_2_ substrate.

## 2. Materials and Methods

### 2.1. ZrO_2_ Substrate Preparation

A set of fifteen rectangular ZrO_2_ blocks were obtained by employing a cutting technique on pre-sintered yttrium-stabilized zirconium dioxide blanks (IPS e.max ZirCAD; Ivoclar Vivadent, liechtenstein) with the use of a saw blade (Isomet 5000; Buehler Ltd., IL, USA). The dimensions of the blocks were measured to be 42 mm in length, 20 mm in width, and 7 mm in height. Subsequently, the ZrO_2_ blocks underwent the sintering process in a precisely calibrated furnace (Programat S1; Ivoclar Vivadent, liechtenstein), adhering to the prescribed instructions provided by the manufacturer. To achieve surface preparation, a wet-grinding technique was employed, utilizing 600-grit silicon-carbide abrasive paper. The specimens were then subjected to a period of immersion in distilled water for 5 min, before using an ultrasonicator for thorough cleaning and finally allowing specimens to air-dry.

### 2.2. Surface Treatment of ZrO_2_ Blocks

The control group (G_0_) was assigned no intervention or treatment. Conversely, the TBC group (G_1_) underwent a procedure in which the upper sections of the specimens were coated with silica using Rocatec Soft powder (3M ESPE, Seefeld, Germany). The powder contained silica-modified aluminum oxide particles with a particle size of 30 μm. The coating process entailed consistent rotary movements in a jet at a pressure of 280 bar for a duration of 15 s, covering an area of 1.0 cm^2^, in accordance with the manufacturer’s instructions. The silica-coating procedure was consistently performed by the same operator, who maintained a fixed distance of 10 mm from the ZrO_2_ specimens. Subsequently, the specimens underwent ultrasonic cleaning in distilled water for 10 min and were air dried. Then, a single coat of a commercially available universal primer, Monobond S (Ivoclar Vivadent, Liechtenstein), was applied using a micro brush and allowed to dry for 5 min in ambient air.

The ZrO_2_ specimens in groups G_2_, G_3_, and G_4_ underwent irradiation using a Femtosecond laser beam, facilitated by a spherical focusing lens with a numerical aperture of 39. A single pass of femtosecond laser pulses, characterized by a wavelength of 1026 nm, pulse duration of 220 fs, and a repetition rate of 100 kHz, was employed to create patterns on the specimens’ bonding surface. The fabrication process involved maintaining a fixed scan speed of 5 mm/s and a laser fluence of 160 J/cm^2^. The specific descriptions of the different patterns within G_2_, G_3_, and G_4_ groups can be found in Table 1.

Following the completion of surface treatment procedures, the ZrO_2_ specimens in all groups underwent an additional round of ultrasonic cleaning in distilled water for 10 min to ensure the removal of any contaminants. To maintain coherence and accuracy in the obtained results, all experimental protocols were meticulously followed and executed by a singular operator throughout the entire process.

### 2.3. Surface Roughness Evaluation

To gauge the effect of surface treatment on ZrO_2_ specimens, a 3D optical non-contact surface profiler (Contour GT, Bruker, CA, USA) was used. The roughness average parameter (Sa) was selected as the metric for analyzing the surface’s amplitude properties. Utilizing non-contact scanning white light interferometry with a standard objective camera featuring a 5× magnification, the instrument was positioned on a vibration isolation table. The profiler scanned all areas of the block where resin stub samples were intended to be bonded (n = 10/group). Each scanned region measured approximately 1.3 mm by 1.0 mm and corresponded to the central area where the resin stubs samples were bonded. The mean surface roughness for the specific group was determined by calculating the average value of the ten readings. To control the precision and measurements of surface roughness parameters, the Vision64 (v 5.30) application software (Bruker, Campbell, CA, USA) was utilized.

### 2.4. Water Contact Angle Measurement

The pretreated ZrO_2_ blocks were evaluated for water contact angle measurement using a camera-based optical tensiometer (Theta Lite, Dyne Technology, Staffordshire, UK). With a syringe tip, a 3.0-μL droplet of distilled H_2_O was applied to wet the specimen surface and placed on a movable table. The contact angle was measured after 30 s when the droplet was stabilized. The illuminated drop was captured from the opposite side by the camera. The contact angle was then automatically calculated by the computer connected to the optical tensiometer.

### 2.5. Surface Morphology Test

The surface-treated ZrO_2_ specimens were evaluated for morphologic examination using a scanning electron microscope (SEM, JEOL, JSM 5900LV, Tokyo, Japan). A single ZrO_2_ specimens from each group having the dimensions of 5 mm × 5 mm × 2 mm was gold sputtered and examined at 20-kV acceleration and 100× magnification.

### 2.6. Preparation of Resin Composite Stubs on ZrO_2_ Specimens

A silicon mold with an inner diameter of 4.0 mm and a height of 4.5 mm was securely positioned on the ZrO_2_ substrate to create a bonded resin stub. To ensure optimal adhesion and minimize air bubbles, a manual instrument was utilized during the process to establish proper contact at the interface between the resin composite and the ZrO_2_ surface. The resin stubs were bonded and subjected to light-curing on each ZrO_2_ specimens (n = 10 per block). The light-curing procedure involved applying light from the top for 40 s, followed by an additional 40 s of light-curing from the lateral sides, utilizing a light-curing unit (Elipar™ 2500, 3M ESPE, St. Paul, MN, USA) with an output power of 600 mW/cm^2^. Subsequently, all ZrO_2_ specimens with bonded resin stub specimens were stored at room temperature for 24 h.

### 2.7. Storage Protocol

A subset comprising one-third of the specimens, specifically 10 resin composite stubs, from each study group was subjected to enclosed mold shear bond strength (EM-SBS) testing to establish the baseline values. The remaining two-thirds of the specimens underwent artificial ageing in a thermocycling device (Model 1100, SD Mechatronik, Feldkirchen-Westerham, Germany) for either 2500 or 5000 cycles. The thermocycling process involved alternating immersion in distilled water between temperatures of 5 °C and 55 °C. Each bath had a dwell time of 10 s, with a transfer time of 10 s between the baths.

### 2.8. EM-SBS Test

The resin composite stubs adhered to the ZrO_2_ blocks were engaged perpendicularly to the chisel blade with blunt edge and loaded onto a universal testing machine (Model no. 3369 Instron, Canton, MA, USA) with a cross head speed of 2 mm/min until failure. The EM-SBS was calculated using the following equation:

S = L/A(1)

where L = applied load at failure in newton (N), and A = adhesive area of the specimen in mm^2^.

### 2.9. Fractographic Investigation

Fractured or debonded specimen surfaces were examined using a stereomicroscope (Nikon SM2-10, Tokyo, Japan) at 15× magnification. The fractured surfaces were assigned to the following three failure patterns: cohesive failure is when the failure is within the resin composite; adhesive failure is when the failure is at the resin composite/ZrO_2_ interface; or mixed failure, i.e., having both adhesive and cohesive failure modes.

### 2.10. Statistical Analysis

The Shapiro–Wilk test was utilized to assess the normal distribution of the data. The statistical differences between the study groups for surface roughness and contact angle were evaluated by one-way analysis of variance (ANOVA). For the analysis of EM-SBS, a two-way ANOVA was utilized. To identify pairwise differences among the groups, Tukey’s post hoc test was applied, employing a multiple comparison procedure. All statistical analyses were conducted using SPSS version 28.0 (SPSS, Chicago, IL, USA), with a significance level set at *p* < 0.05.

## 3. Results

**Figure 1:**
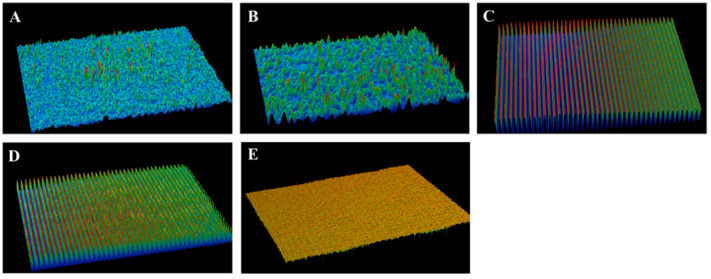
Three-dimensional pictograms of surface roughness measurements on the ZrO_2_ blocks. (**A**–**E**) depict the ZrO_2_ specimens of G_0_, G_1_, G_2_, G_3_, and G_4_, respectively.

**Figure 2:**
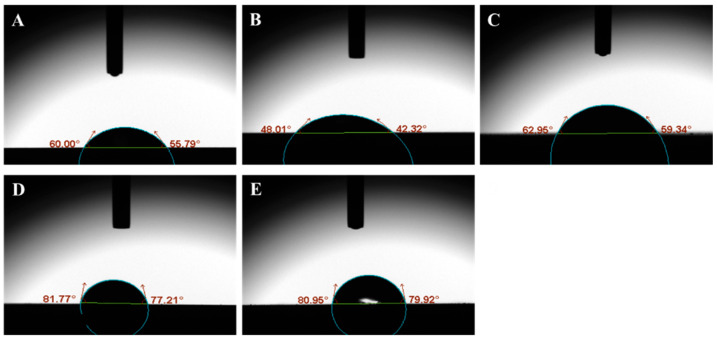
Pictograms of water contact angle formation on the ZrO_2_ specimens. (**A**–**E**) depict the ZrO_2_ blocks of G_0_, G_1_, G_2_, G_3_, and G_4_, respectively.

Table 2 presents the measurements of surface roughness (expressed in µm) and water contact angle (expressed in degrees) on the treated ZrO_2_ specimens within the various study groups. The group denoted as G_4_ exhibited the highest surface roughness, measuring 23.05 ± 3.51 µm, whereas the control group (G_0_) displayed the lowest surface roughness, specifically 1.65 ± 0.53 µm. Conversely, the G_3_ group demonstrated the highest water contact angle formation on the ZrO_2_ substrate, measuring 79.56 ± 1.41 degrees, while the G_1_ group displayed the lowest water contact angle formation, measuring 47.91 ± 2.01 degrees. Figure 1 and Figure 2 further elaborate the achieved results of surface roughness and contact angle measurements, respectively.

**Figure 3:**
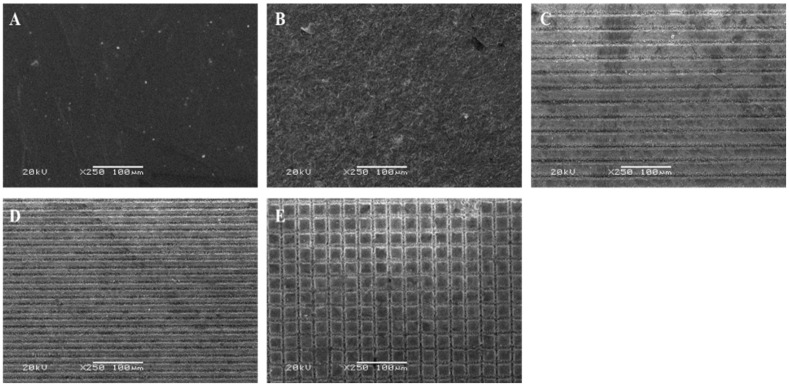
SEM pictograms of study groups using different surface treatments: (**A**–**E**) depict the ZrO_2_ blocks of G_0_, G_1_, G_2_, G_3_, and G_4_, respectively.

Figure 3 represents the SEM pictograms of ZrO_2_ blocks treated with various methods. Diverse roughness patterns were observed at the SEM evaluation of conditioned surfaces. The G_0_ group (Figure 3A) exhibited a smooth and polished ZrO_2_ surface while G_1_ showed an irregular morphological pattern (Figure 3B). Among the femtosecond laser-treated ZrO_2_ specimens, a well-defined pattern can be observed of deep horizontal grooves 30 µ m apart (Figure 3C) and 15 µm apart (Figure 3D) in G_2_ and G_3_ groups, respectively. A well-defined pattern of deep cross grooves 30 µ m apart also can be observed in the G_4_ group (Figure 3E).

Table 3 displays EM-SBS values of the study groups. Significant differences (*p* < 0.05) were observed in the mean EM-SBS values among the groups at baseline, 2500 thermocycles, and 5000 thermocycles. The highest mean EM-SBS was observed in G_4_ at baseline (17.57 ± 3.01 MPa). The lowest mean EM-SBS was observed in the G_0_ group that underwent 5000 thermocycles (5.47 ± 0.97 MPa). At the end of 2500 and 5000 thermocycles, G_4_ exhibited statistically significant differences from G_0_ and G_2_ groups. All tested groups exhibited a reduction in EM-SBS due to artificial water ageing.

**Figure 4:**
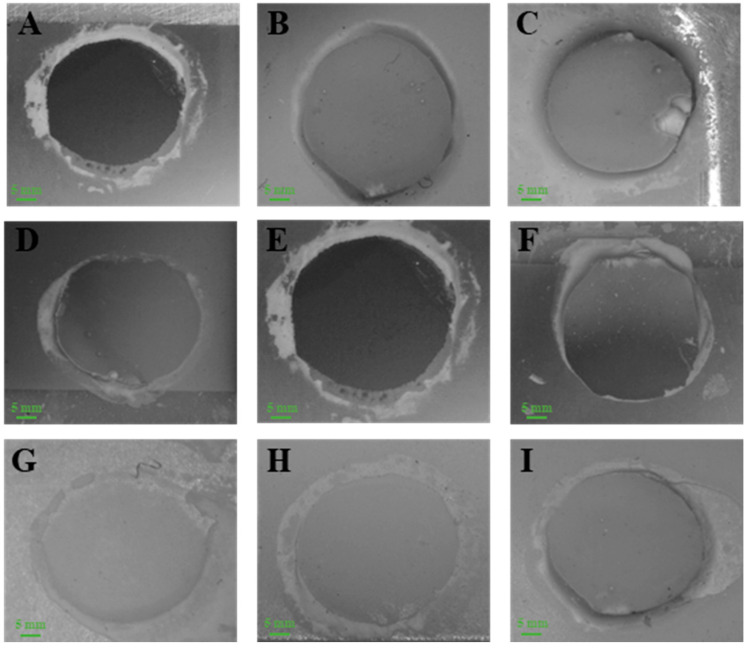
Stereomicroscopy images of the study samples, depicting various failure modes in different groups. In the baseline groups, (**A**) shows adhesive failure in G_0_, (**B**) displays cohesive failure in G_1_, and (**C**) demonstrates cohesive failure in G_2_. Among the groups subjected to 2500 thermocycles, (**D**) exhibits mixed failure in G_1_, (**E**) reveals adhesive failure in G_3_, and (**F**) presents mixed failure in G_4_. In the 5000 thermocycles groups, (**G**) portrays adhesive failure in G_2_, (**H**) showcases mixed failure in G_1_, and (**I**) exhibits cohesive failure in G_4_.

Furthermore, the failure mode analysis is reported in Table 3. At baseline, the predominant failure mode was adhesive; however, mixed and cohesive failure modes were observed among the groups G_1_, G_2_, G_3_ and G_4_. The thermocycling had a deleterious effect, particularly in the G_0_ group, where 10% and 30% resin stub samples showed spontaneous debonding after 2500 and 5000 cycles, respectively. Meanwhile, the percentage of mixed and cohesive failures was reduced among the study groups. Due to artificial water ageing, none of the groups exhibited cohesive failure mode at the end of 5000 thermocycles except G_4,_ where 10% failure was observed as cohesive. Details can be observed in Table 3 and Figure 4. 

Table 4 presents the two-way ANOVA model of the outcome. The study groups as well as the ageing had a significant effect on the EM-SBS (*p* < 0.001). Moreover, their interactive effect was also observed as significant (*p* < 0.001).

## 4. Discussion

A higher SBS was observed in G_4_ using femtosecond laser irradiation compared to the G_1_ group. However, the difference was insignificant, hence the null hypothesis is rejected. It was noticed that increased surface roughness does not correlate with the formation of a favorable retentive ZrO_2_ surface with resin composite. However, the G_4_ group can be observed as exceptional where the increased surface roughness (23.05 ± 3.51 µm) can explain the enhanced resin composite-ZrO_2_ bonding. The reason could be the engraving nature of patterns, i.e., the cross stripe pattern that created retentive irregularities [13] and hence increased the SBS of the resin composite to ZrO_2_. In contrast, the surface roughness of the TBC group (i.e., G_1_ group) showed statistically lower values against all the femtosecond laser-treated ZrO_2_ groups (i.e., G_2_, G_3_ and G_4_). However, the observed SBS of G_1_ was either statistically higher or at par with femtosecond laser-treated ZrO_2_ groups. This may be due to many interrelated influencing factors, such as mechanical and chemical pretreatment methods, surface contamination, compatibility of silane primers with ZrO_2_ substrate and storage conditions. The findings of the present investigation are in line with the previous studies that advocated consistent and profound surface roughness with the use of the femtosecond laser treatment [13,14].

The contact angle measurement is an important parameter in defining the physical surface features. The lower water contact angle suggests that the adhesive on the surface would effectively wet the substrate surface, which is one of the key factors for improved bonding [15]. From the present data, a correlation of contact angles and surface roughness could not be established. This may suggest that the surface roughness parameter has no effect on the wettability of the adhesive on the substrate. The lower water contact angle in G_1_ specimens having a lower surface roughness (i.e., 2.44 ± 0.37 µm) suggest that TBC along with silane primer might adequately wet the methacrylate-based resin composite to the pretreated ZrO_2_ surface. Among the femtosecond laser groups, it was observed that the ultrafast laser induces various structural and chemical changes on the ZrO_2_ substrate leading to hydrophobic surfaces with increased water contact angles’ formation. The results are in line with the previous study that advocated increased water contact formation compared to TBC on ZrO_2_, depending on the engraving nature of pattern [16].

The surface topographic evaluation using SEM suggest substantial quality changes in the ZrO_2_ surface after femtosecond laser irradiation. SEM pictograms confirms that femtosecond laser irradiation is an effective method for topographic changes on ZrO_2_ substrates [17]. Among the laser irradiation groups, we observed well-structured, regular and well-defined horizontal and cross surface patterns without any visible microcrack formation (Figure 3C–E). The use of ultrashort laser pulses could avoid thermal and mechanical stresses [18]. In contrast, the TBC-treated surface produced a rough, irregular surface with intermittent microcracks (Figure 3B) [19]. This study is in line with another study by Kara et al. that demonstrated the irradiation efficiency of the femtosecond laser for roughening the surfaces of ZrO_2_ [20].

Furthermore, SBS in this study was evaluated using an enclosed mold, as it is a promising approach for the reliability of the results. Five different surface pretreatments were evaluated with significant differences in EM-SBS values. However, the combination of silica coating and application of silane primer on ZrO_2_ is considered an essential and widely used method for improved resin composite to ZrO_2_ bonding [3]. On the other hand, the mechanical bonding relies heavily on the surface roughness and texture of the ZrO_2_ surface [3,21]. Surface roughness is an important parameter for mechanical bonding [3]. However, it is notable that we observed that higher surface roughness does not warrant higher resin composite to ZrO_2_ bonding, as observed in groups G_2_ and G_3_. In the case of the G_4_ group, the ZrO_2_ substrate was pretreated with precise micropatterning, and the creation of cross retention microgrooves of 30 μm apart produced the highest EM-SBS, irrespective of the ageing condition used. The reason for this could be the highest surface roughness (i.e., 23.05 ± 3.51 µm) that provided a greater surface area to which the resin composite could adhere. The resin composite can flow into the irregularities of the rough surface, creating a more intimate contact and interlocking with the ZrO_2_ substrate. The findings of this study contradicts with the previous investigators who advocated for a correlation between the SBS and surface roughness of the ZrO_2_ substrate [13]. The lowest EM-SBS in the control group was due to the polished and unreactive surface of ZrO_2_. However, the lower EM-SBS in G_2_ and G_3_ (with horizontal lines) might hint that the orientation of the micropattern lines can significantly affect the surface topography. Horizontal lines may create a smoother and less retentive surface compared to cross lines. It is equally possible that the orientation of the micropattern can influence the stress distribution within the resin composite to the ZrO_2_ interface. Due to the concentration of stress in one direction, when a shear force is applied, the frictional resistance along the sliding plane may not be sufficient and hence bond strength is affected. Meanwhile, the cross lines may distribute stresses more evenly, reducing the likelihood of bond failure. 

In contrast to the initial baseline readings, the results of the EM-SBS following exposure to water ageing exhibited a noteworthy decline. The outcomes derived from this investigation imply that the bond between the resin composite to ZrO_2_ was susceptible to hydrolytic degradation, irrespective of the employed surface pretreatment methodologies. The observed reduction in the SBS values aligns with the findings of the earlier investigators [4].

The stereomicroscope images (in Figure 4) showed a predominately adhesive failure mode among the study groups. Among baseline readings, 20% of the failure modes were reported as mixed in both G_1_ and G_4_ groups, suggesting the effectiveness of the pretreatment methods. At the end of 5000 thermocycles, 20% was a mixed failure in the G_1_ group while there were 10% cohesive and 10% mixed failure modes in G_4_. These findings strongly indicate the durability of the bond and reliability of the pretreatment methods. The higher percentage of mixed and cohesive failures in G_4_ might be attributed to increased wettability and contact area for mechanical interlocking in the G_4_ group [14]. This study aligns closely with the results reported by Atsu et al., wherein adhesive failures were documented at lower bond strength values, while higher bond strengths were associated with mixed and cohesive failures [22].

In the current study, only one type of resin composite was used, therefore it can be considered as one of the limitations. The compositional variations in the resin composite may affect the bonding strength; hence, it could be suggested to evaluate multiple resin composite systems in further works. A static mechanical test for the bond strength evaluation was used; therefore, the use of cyclic fatigue evaluations would best mimic the oral environment. 

## 5. Conclusions

In the context of this laboratory investigation, the following conclusions were derived:

- The femtosecond laser-treated ZrO_2_ specimens showed statistically higher surface roughness values than the G_0_ and G_1_ groups. However, the roughness depends on the laser scanning pattern.
- Femtosecond laser irradiation generates structural and chemical changes on the ZrO_2_, leading to increased water contact angle formation.
- Well-structured, regular and well-defined micropatterns are possible without any visible microcrack on ZrO_2_ using the femtosecond laser irradiation.
- Not all the micropatterns of the femtosecond laser are effective in improving the EM-SBS between the resin composite and ZrO_2_.
- The EM-SBS of G_4_ group (i.e., with cross lines, 30 µm apart) was observed highest at baseline as well as at the end of 2500 and 5000 thermocycles.

## Figures and Tables

**Table 1:** Tested groups and their surface pretreatment methods.

Group	Wet-Grinding	Surface Treatment
G_0_	600-grit SiC abrasive paper	No treatment
G_1_	600-grit SiC abrasive paper	Tribochemical silica coating with 30 µm silica-coated Al_2_O_3_ powder
G_2_	600-grit SiC abrasive paper	Horizontal lines with femtosecond laser ablation, 30 µm apart
G_3_	600-grit SiC abrasive paper	Horizontal lines with femtosecond laser ablation, 15 µm apart
G_4_	600-grit SiC abrasive paper	Cross lines with femtosecond laser ablation, 30 µm apart

**Table 2:** The surface roughness and water contact angle measurements of ZrO_2_ blocks after various treatments.

Group	Surface Roughness (S_a_, µm)	Contact Angle (°)
G_0_	1.65 ± 0.53 _a,b,c_	58.28 ± 3.75 _a,b,c_
G_1_	2.44 ± 0.37 _d,e,f_	47.91 ± 2.01 _a,d,e,f_
G_2_	11.52 ± 1.22 _a,d,g,h_	62.37 ± 1.73 _d,g,h_
G_3_	17.68 ± 4.68 _b,e,g,i_	79.56 ± 1.41 _b,e,g_
G_4_	23.05 ± 3.51 _c,f,h,i_	78.73 ± 1.74 _c,f,h_

Key: The lower-case subscript letters denote significant differences between the groups.

**Table 3:** Enclosed mold shear bond strength (EM-SBS) values recorded in the study groups and their corresponding failure mode after shear testing.

Group (n = 10)	EM-SBS (MPa) (Mean ± SD)			Failure Mode Analysis (%)			
	24 h	2500 Cycles	5000 Cycles	DE	AD	CO	MI
	9.51 ± 1.95 _a,b,c,d_			0	100	0	0
G_0_		7.87 ± 1.66 _g,h,i_		10	90	0	0
			5.47 ± 0.97 _m,n,o,p_	30	70	0	0
	15.97 ± 2.65_a,_			0	70	10	20
G_1_		14.18 ± 1.76 _g,j_		0	80	0	20
			13.80 ± 3.01 _m,q_	0	80	0	20
	12.90 ± 2.90 _b,e_			0	90	0	10
G_2_		9.48 ± 1.68 _j,k,l_		0	100	0	0
			8.47 ± 0.94 _n,q,r,s_	10	90	0	0
	13.01 ± 2.21 _c,f_			0	90	0	10
G_3_		12.55 ± 1.28 _h,k_		0	90	0	10
			12.05 ± 1.91 _o,r_	0	90	0	10
	17.57 ± 3.01 _d,e,f_			0	60	20	20
G_4_		14.49 ± 2.00 _i,l_		0	70	10	20
			14.05 ± 4.21 _p,s_	0	80	10	10

Key: The same lower-case alphabets depict statistically significant differences between the storage groups. DE: Debonding; AD: Adhesive failure; CO: Cohesive failure; MI: Mixed failure.

**Table 4:** Two-way analysis of variance model of the study groups and their storage conditions.

Source	Type III Sum of Squares	df	Mean Square	F	Sig.
Corrected Model	1510.487	14	107.892	22.667	0.000
Intercept	21,915.460	1	21,915.460	4604.289	0.000
Group	236.652	2	118.326	24.860	0.000
Ageing	1218.577	4	304.644	64.004	0.000
Group×Ageing	55.257	8	6.907	1.451	0.181
Error	642.572	135	4.760		
Total	24,068.519	150			
Corrected Total	2153.059	149			

## Data Availability

Not applicable.
